# A Four-Feet Walking-Type Rotary Piezoelectric Actuator with Minute Step Motion

**DOI:** 10.3390/s18051471

**Published:** 2018-05-08

**Authors:** Yingxiang Liu, Yun Wang, Junkao Liu, Dongmei Xu, Kai Li, Xiaobiao Shan, Jie Deng

**Affiliations:** State Key Laboratory of Robotics and System, Harbin Institute of Technology, Harbin 150001, China; 1120810102@hit.edu.cn (Y.W.); jkliu@hit.edu.cn (J.L.); dongmeixu@hit.edu.cn (D.X.); 14B908004@hit.edu.cn (K.L.); shanxiaobiao@hit.edu.cn (X.S.); 16b908017@stu.hit.edu.cn (J.D.)

**Keywords:** rotary piezoelectric actuator, four feet walking, rectangular motion, bending motion

## Abstract

A four-feet walking-type rotary piezoelectric actuator with minute step motion was proposed. The proposed actuator used the rectangular motions of four driving feet to push the rotor step-by-step; this operating principle was different with the previous non-resonant actuators using direct-driving, inertial-driving, and inchworm-type mechanisms. The mechanism of the proposed actuator was discussed in detail. Transient analyses were accomplished by ANSYS software to simulate the motion trajectory of the driving foot and to find the response characteristics. A prototype was manufactured to verify the mechanism and to test the mechanical characteristics. A minimum resolution of 0.095 μrad and a maximum torque of 49 N·mm were achieved by the prototype, and the output speed was varied by changing the driving voltage and working frequency. This work provides a new mechanism for the design of a rotary piezoelectric actuator with minute step motion.

## 1. Introduction

In the field of modern precision operation, micro-nano operation has become the key technology; there are strong demands for actuators with high resolutions. Piezoelectric actuators usually convert electrical energies into mechanical energies by using the inverse piezoelectric effects and frictional forces [1,2,3]. They have simple structures, fast responses, low speeds, self-locking when powered off, and high positioning accuracies, which give them wide application prospects in robot joints, optical instruments, MEMS, positioning stages, and space mechanisms [4,5,6,7,8,9].

Piezoelectric actuators can work at a resonant state [10,11,12,13,14] or non-resonant state [15,16,17,18,19,20,21]. From the viewpoint of the connecting form of the piezoelectric element and the stator, the piezoelectric actuators can also be divided into the bonded-type and clamped-type. A new bonded-type piezoelectric driving micro-robot based on bending-bending resonant vibration was introduced by Su et al., the load capacity and speed were 200 g and 33.45 mm/s, respectively [22]. Yan et al. proposed a three-DOF ultrasonic motor using four piezoelectric ceramic plates in a bonded-type structure, the output velocities of three DOF can reach 280 rpm, 277 rpm, and 327 rpm, respectively [23]. Yu et al. proposed a U-shaped clamped-type linear piezoelectric ultrasonic motor using longitudinal transducers [24], the maximum output speed and thrust force with the 100 V_rms_ were 416 mm/s and 21 N, respectively. Comparing with the bonded-type resonant piezoelectric actuators, the clamped-type resonant ones have advantages of high speed and large stroke, but their positioning accuracies are usually on micrometer scales. Kazumasa et al. proposed a V-shape transducer ultrasonic motor, which has three operation modes: a fast motion mode driven by a single-phase resonance frequency, a nanometer motion mode driven by DC voltage, and an inertial driving mode using a low-frequency saw-wave voltage for unlimited stroke motion in DC driving; under the non-resonance motion modes, the resolution of the motor can reach nanometer scales [25].

The non-resonant piezoelectric actuators are suitable for a precision positioning system for its high resolution, rapid response, and low energy consumption advantages [26,27]. Based on the working mechanism, the non-resonant piezoelectric actuators can be divided into the direct-driving [28,29], inertial-driving [30,31,32], and inchworm-type [33,34,35] actuators. The direct-driving actuators operate using the axial direction deformations of the PZT stacks, they can achieve nanometer positioning easily along the axial direction under the *d*_33_ working mode. However, the direct output displacement of the PZT stack is usually small, which is approximately 0.1% of the total thickness. Thus, mechanical magnification mechanisms, like the combination of levers and flexure hinges, are utilized to magnify the output displacement. Clark et al. designed a flexure-based precision positioning stage, their prototype achieved a rotary resolution of 0.075 μrad and a rotary stroke of 535.8 μrad [18]; Juuti et al. designed a bridge-type amplifier structure to amplify the displacement of the input translation [28]; Li et al. compounded the parallelogram flexures and bridge-type displacement amplifiers in an XY stage [29]; meanwhile, the use of the magnification mechanism decreases the output stiffness of the actuator, making the output force relatively small. Inertia-driving actuators, based on the inertia theorem, utilize the interaction between the stator and mover to produce the precision motion. The inertia-driving actuators have flexible structures, the principles of which are simple. Wang et al. proposed a novel inertia-driving rotary actuator based on compliant foot driving, and their prototype produced a rotary resolution of 0.24 μrad [19]. Li et al. designed a dual-servo nano-positioning stage, and it achieved a resolution of 1.54 μrad [20]. Nomura et al. developed an inertia-driven micro robot using four PZT stacks, the differences between the static frictional force and the impulsive inertial force caused by the rapid deformations of the PZT elements were used for the movement [32]. Due to the working mechanism, the driving forces of the inertia-driving actuators are relatively small; additionally, the demands of the driving signals are strict. The inchworm-type actuators are inspired by the mechanism of the inchworm, which usually include the driving component and the clamping component; they usually have large output forces and long strokes. Kim et al. introduced a hybrid linear actuator under the inchworm motion principle [33]; Moon et al. developed a fast inchworm type actuator [35], which indicated that the positioning accuracy of the inchworm actuators can reach the nanometer level and the output speed was usually less than 1 mm/s. However, the inchworm-type actuators usually have complex structures and strict demands of manufacture accuracy. In addition, most of the existing precision piezoelectric actuators are of the linear type; there are very few precision rotary piezoelectric actuators, which limits the application of the non-resonant piezoelectric actuators. Furthermore, they also have problems of low resolutions or limited strokes. Therefore, there is a great deal to do to develop the rotary precision actuators. 

The precise rotary motion is achieved by a piezoelectric actuator with large stroke and high resolution in this study. A novel four-feet walking mechanism based on the rectangular trajectory is proposed, in contrast to the traditional direct-driving, inertial-driving, and inchworm-type actuators. Two orthogonal bending motions are used to form the rectangular motion on each driving foot; a rotary stepping motion of the rotor is accomplished by the cooperation of four driving feet moving in rectangular trajectories. 

## 2. Structures and Principles 

**Figure 1:**
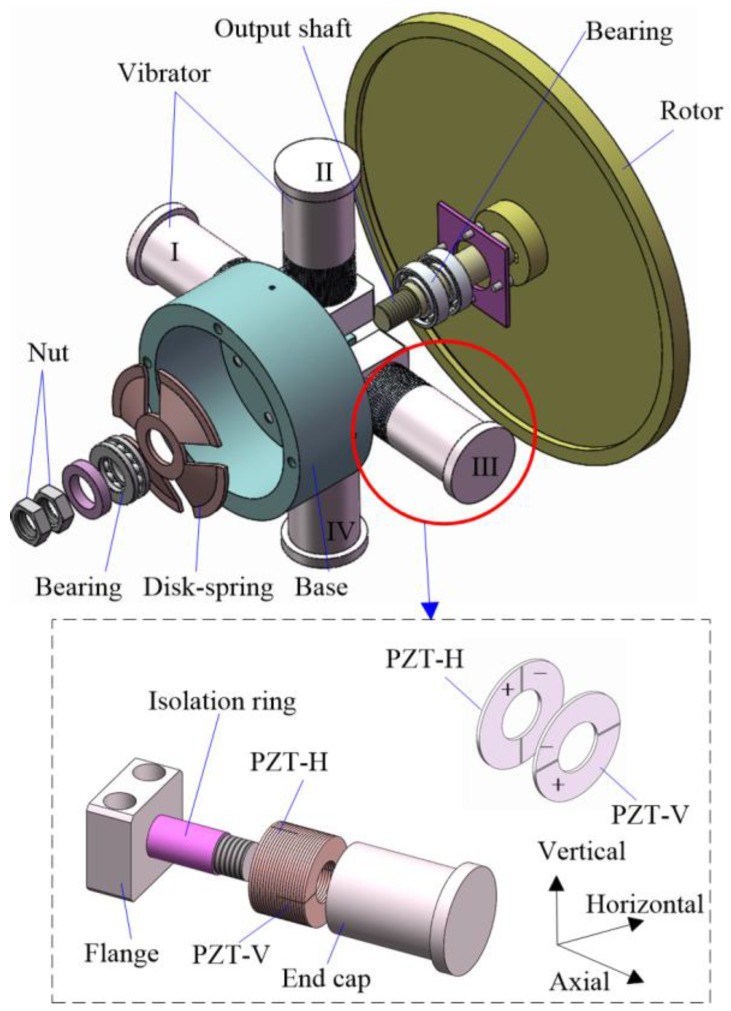
Three-dimensional structure of the piezoelectric actuator.

The structure of the proposed actuator is shown in Figure 1. It is comprised of a stator, a rotor, and an output shaft; the stator contains a base and four bending vibrators with the same structure. A nut-disk-spring system is used to apply the preload between the driving feet and the rotor. Each vibrator is made up of one end cap, two groups of PZT elements, and one flange bolt, and the PZT elements are clamped between the flange and the end cap by a bolt. The tip of the end cap serves as the driving foot. Twenty pieces of PZT ceramic rings with an outer diameter of 30 mm and thickness of 1 mm are divided into two groups to excite the horizontal and vertical bending deformations, and they are named PZT-H and PZT-V respectively. Each PZT ring contains two half-ring partitions with inverse polarizations, which are noted with “+” and “−”. Each vibrator has a total length of 80 mm. Beryllium bronze sheets are clamped between each PZT ceramic to serve as electrodes. A plastic ring is set between the electrodes and the bolt to achieve the isolations of the electrodes.

**Figure 2:**
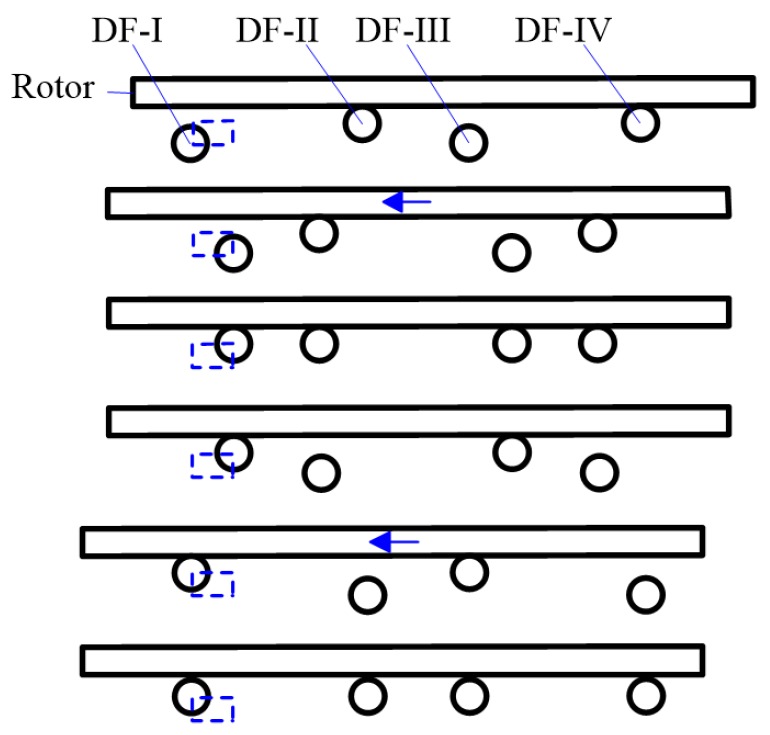
Illustration of the four-feet walking mechanism (expansion view along circumferential direction).

The working mechanism of the proposed piezoelectric actuator is shown in Figure 2, in which the four driving feet are marked as DF-I to DF-IV, respectively. The rotor is pushed by the static friction between it and the driving feet. The driving foot will bend upwards and lock the rotor when a positive direct current (DC) voltage is applied on PZT-V, while the driving foot will bend downwards when a negative DC voltage is applied. In a similar way, the driving foot will bend leftwards when a positive DC voltage is applied on PZT-H, and the driving foot will bend rightwards if a negative DC voltage is applied.

The motion sequences of the four driving feet are as follows:

(1) DF-I and DF-III bend downward and depart from the rotor, DF-II and DF-IV lock the rotor.
(2) DF-I and DF-III bend rightward, DF-II and DF-IV bend leftward synchronously and push the rotor leftward for one step.
(3) DF-I and DF-III bend upward to lock the rotor.
(4) DF-II and DF-IV bend downward and depart from the rotor, DF-I and DF-III remain locking the rotor.
(5) DF-I and DF-III bend leftward and push the rotor leftward for another step, DF-II and DF-IV bend rightward synchronously.
(6) DF-II and DF-IV bend upward to lock the rotor.
(7) Repeat (1)–(2)–(3)–(4)–(5)–(6).

**Figure 3:**
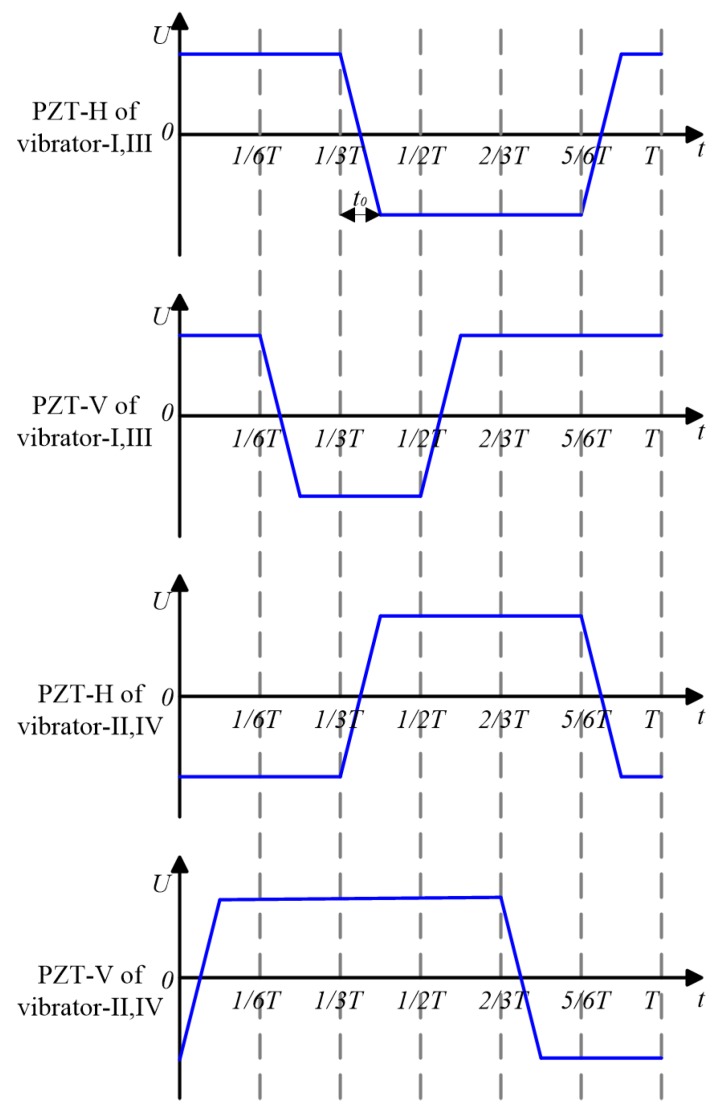
The driving signals applied on the proposed actuator.

Therefore, the driving feet will move along rectangular motion trajectories with special temporal shifts and the rotor will be pushed leftward for two steps in one cycle. A large stroke can be accomplished through the accumulations of small steps. Four driving signals are needed to achieve the above motion sequences: the two signals applied on the PZT elements of vibrator-I and vibrator-III are the same, so as the two signals applied on the PZT elements of vibrator-II and vibrator-IV, as shown in Figure 3. Here, *T* is the time of one cycle; *t*_0_ is the transitional time during the variation process of voltage amplitude. The rotor will be pushed rightward by adjusting the temporal shift of the driving signals.

## 3. Simulation Analyses

**Figure 4:**
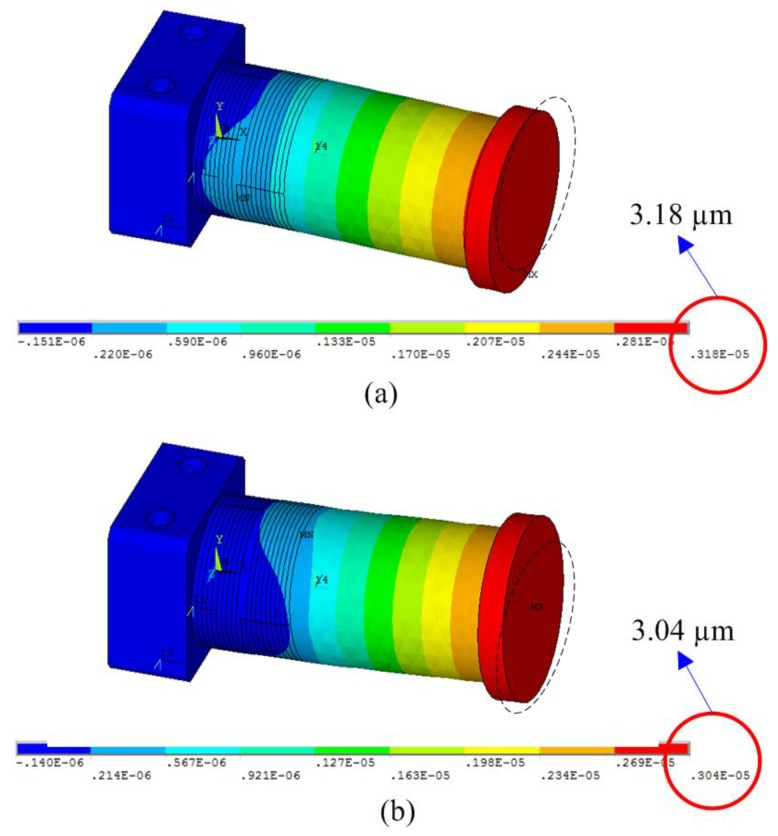
The static analysis results. (**a**) Horizontal bending deformation. (**b**) Vertical bending deformation.

Firstly, the bending displacements of the vibrators under DC voltages are calculated through ANSYS software (Canonsburg, PA, USA) with static analysis. During the simulation, the material of the flange bolt is set as steel with a density of 7800 kg/m^3^, a Poisson’s ratio of 0.30, and a modulus of elasticity of 206 GPa; the material of the end cap is set as duralumin with a density of 2810 kg/m^3^, a Poisson ratio of 0.33, and a modulus of elasticity of 72 GPa; the PZT elements are set as PZT-4, the parameters of which are listed in Table 1. Fixed boundaries are applied on the top and bottom surfaces of the flange. The bending displacements of the driving foot in horizontal and vertical directions are calculated to be about 3.18 μm and 3.04 μm when DC voltages of 200 V are applied on the PZT-H and PZT-V, respectively, as shown in Figure 4. 

**Figure 5:**
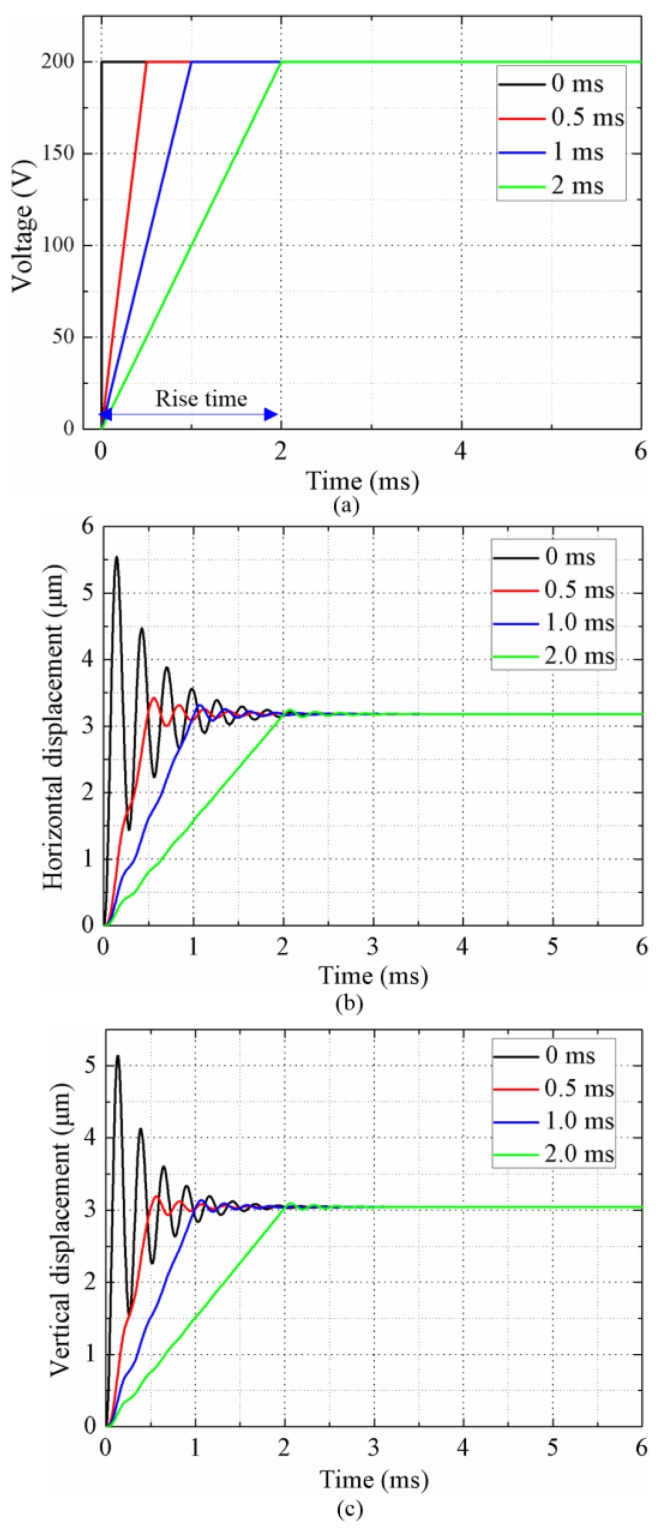
The transient response of the driving foot under step signals with different rise time. (**a**) Driving signals. (**b**) The displacement in horizontal direction. (**c**) The displacement in vertical direction.

Next, the transient response characteristics of the vibrators are obtained through transient analysis. Rayleigh damping (also known as alpha and beta damping) is adopted in the transient analysis. The corresponding damping equation can be represented as:

(1) C=αM+βK

where *αM* represents mass damping, *βK* represents structural damping. The values of *α* and *β* are calculated from the modal damping ratio *ξ* and are required as input parameters for the analysis [36]. The modal damping ratio *ξ* used in the simulation is 0.03, which is obtained from the spectrum map tested by a scanning laser Doppler vibrometer (PSV-400M2, Polytec, Waldbronn, Germany). The transient responses of bending motions in horizontal and vertical directions under step signals with an amplitude of 200 V and rise times of 0 ms, 0.5 ms, 1.0 ms, and 2.0 ms are shown in Figure 5. The steady displacements of bending motions are calculated to be about 3.18 μm in horizontal direction and 3.04 μm in the vertical direction, which are identical to the static analysis results. Oscillations occur at the beginning of response processes and the oscillations decrease with the increase of the rise time of the excitation signal. Thus, the oscillations in the transient response processes can be significantly reduced by extending the rise time of the driving signal, which can make the motions of the driving feet more stable and reduce the slips between the driving feet and the rotor.

The rotor is pushed through the static friction between it and the driving feet, so there will not be slips when inertial force (*F_I_*) is less than the maximum static frictional force (*f_max_*), which can be described as:

(2) $$\mu F=f_{max}\geq F_{I}=\frac{1}{2}\frac{J\ddot{\theta }}{R}$$

(3) $$\ddot{\theta }=\frac{a}{R}$$

Here, *μ* is the static friction coefficient between steel and duralumin (*μ* = 0.3), *F* is the preload, *J* is the moment of inertia of the rotor (*J* = 0.00578 kg·m^2^), $\ddot{\theta }$ is the angular acceleration of the rotor, *a* is the linear acceleration at the edge of the rotor, *R* is the radius of the rotor (*R* = 0.1025 m). When *F* is set as 12 N, The linear acceleration *a* should not be larger than 13.1 m/s^2^ by solving Equations (2) and (3).The maximum linear accelerations of the driving foot are calculated from the transient response results, as shown in Table 2. Thus, there will not be slips if the rise time of the step signal is set as 5 ms under a preload of 12 N.

**Figure 6:**
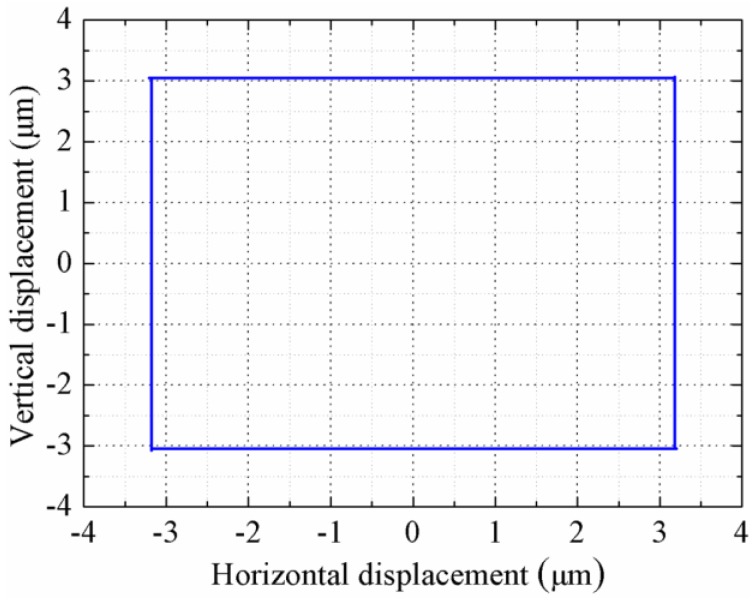
Motion trajectory of the driving foot.

The horizontal and vertical steady displacements are calculated to be about 6.36 μm and 6.08 μm when two trapezoidal signals with a frequency of 1 Hz, an amplitude of 400 V_p-p_, and a rise time of 5 ms are applied on the PZT-H and PZT-V, respectively. The motion trajectory of the driving foot is shown in Figure 6, it is obvious that the foot moves along a rectangular trajectory and there are slight oscillations when the voltage starts to change. It can be predicted that the rotor will be moved along the circumferential direction with 6.36 μm for each step.

## 4. Experiments and Discussion

**Figure 7:**
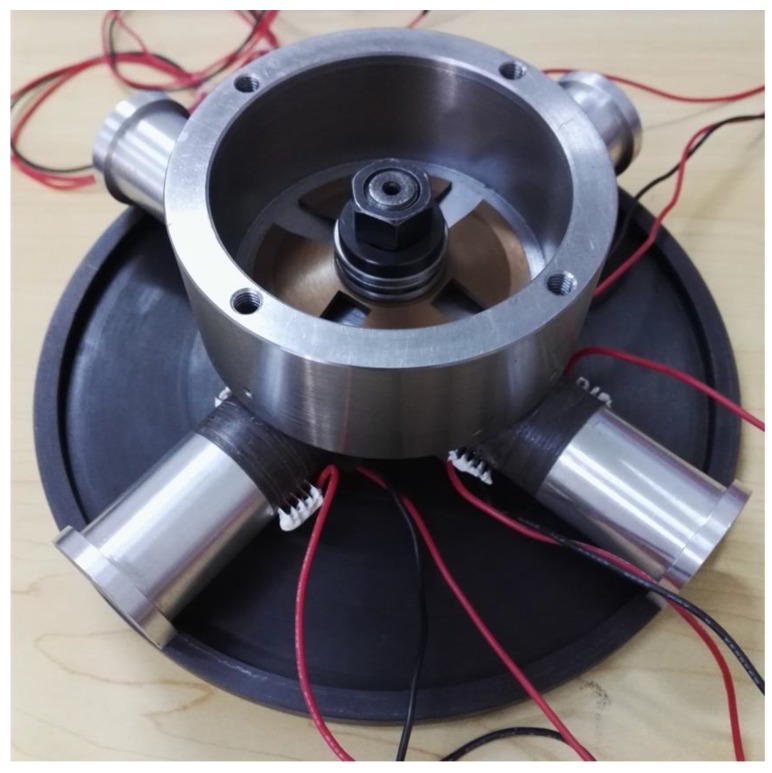
Photo of the prototype.

A prototype was manufactured to verify the feasibility of the proposed actuator, as shown in Figure 7. The average internal capacitance of PZT-H and PZT-V in each vibrator is tested to be 52.25 nF and 49.8 nF, respectively. A laboratory-designed power source was used to provide the excitation signals; its output voltage range, resolution, maximum output current are −210 V~+210 V, 1 V and 150 mA, respectively. A capacitance displacement sensor (PI D-E20.200, PI, Karlsruhe, Germany) with a resolution of 4 nm and a range of 200 μm used to measure the output linear displacement (the linear displacement was approximately equal to the arc length as the angular displacement of the rotor is very small), then the angular displacement and speed were calculated. 

Firstly, the step-displacements (horizontal displacements) of the four vibrators under trapezoidal signals with a frequency of 1 Hz, an amplitude of 400 V_p-p_, and rise time of 5 ms were measured, as shown in Table 3. There are little discrepancies among the horizontal displacements of the four driving feet because of manufacturing errors and assembly errors. From the experimental results we can see that the experimental displacements of the driving feet are in good agreement with the simulation ones (6.36 μm) shown in Figure 6. The very small deviations between the tested displacements and the simulation results are mainly caused by the differences of models, material properties, and boundary conditions between the simulation model and the actual prototype. 

The hysteresis of PZT is a key factor affecting the output displacement of piezoelectric actuators; however, it has little effect on the displacement of the foot in this work because the step displacement of the rotor depends on the horizontal static displacement of the driving feet, and the working frequency is very low (1 Hz in the experiment), which means that the foot has enough time to reach the static state.

**Figure 8:**
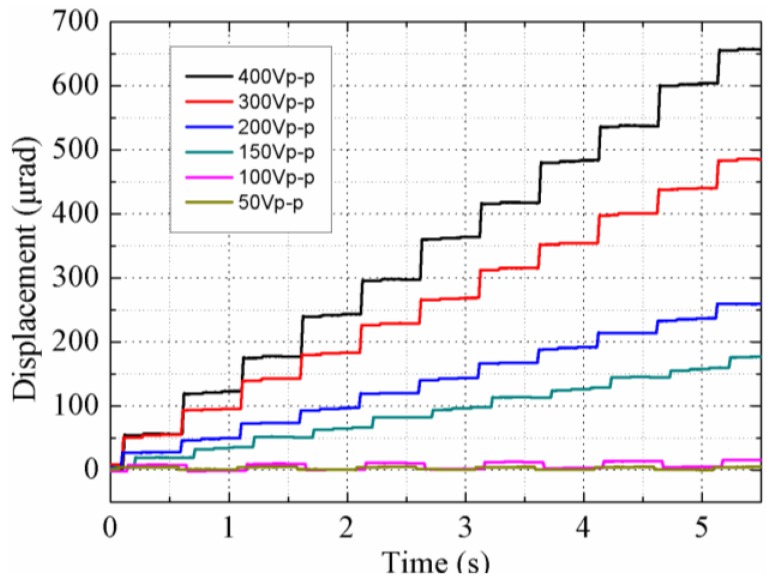
Plot of the output displacement versus the time.

Next, the output displacements of the rotor versus time under different voltages were measured, as shown in Figure 8, during which trapezoidal signals with a frequency of 1 Hz and rise time of 5 ms were used. The rotor is driven twice in one cycle, once by the DF-I and DF-III (Group I) simultaneously (the first step), and another by the DF-II and DF-IV (Group II) simultaneously (the second step). Due to the difference in driving feet’s step-displacements of each group, the step-displacement of the first step is determined by the DF-I with smaller step-displacement, which is approximately 6.27 μm. For the same reason, the step-displacement of the second step is approximately 6.43 μm. The overall average step-displacement is about 6.35 μm, which is consistent with the measured value of 6.32 μm (60.19 μrad). Figure 8 states that the step-displacement can be varied by changing the input voltages, and the rotor is moved with two steps in one cycle. 

**Figure 9:**
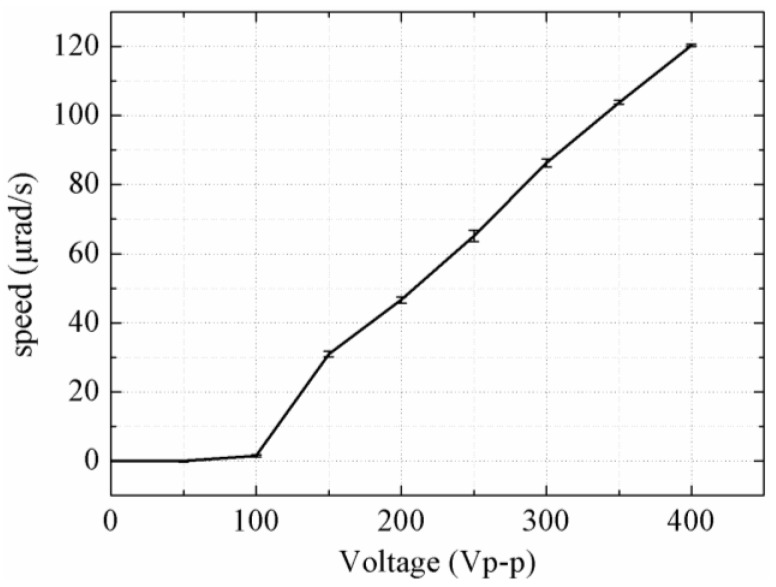
Plot of the speed versus the input voltage.

The rotor is pushed forward with retracement under a voltage of 100 V_p-p_, and it is pushed forward step-by-step under a voltage of 150 V_p-p_ and above. The step-displacements under voltages of 150 V_p-p_, 200 V_p-p_ and 300 V_p-p_ are about 1.15 μm, 2.43 μm and 4.55 μm, respectively. The speed versus the input voltage is plotted in Figure 9, and it can be seen that a maximum speed of 120.38 µrad/s is achieved under a voltage of 400 V_p-p_. The rotor is in a reciprocating motion under input voltages of 50 V_p-p_, as shown in Figure 8, which results in the rotor being unable to move macroscopically. The main reason of the reciprocating motion is that the driving feet cannot depart from the rotor completely under a voltage of 25 V (under the preload of 12 N); thus, the driving feet maintain contact with the rotor during the whole driving cycle; the forward displacement is nearly the same as the backward one under the horizontal voltage shown in Figure 3.

**Figure 10:**
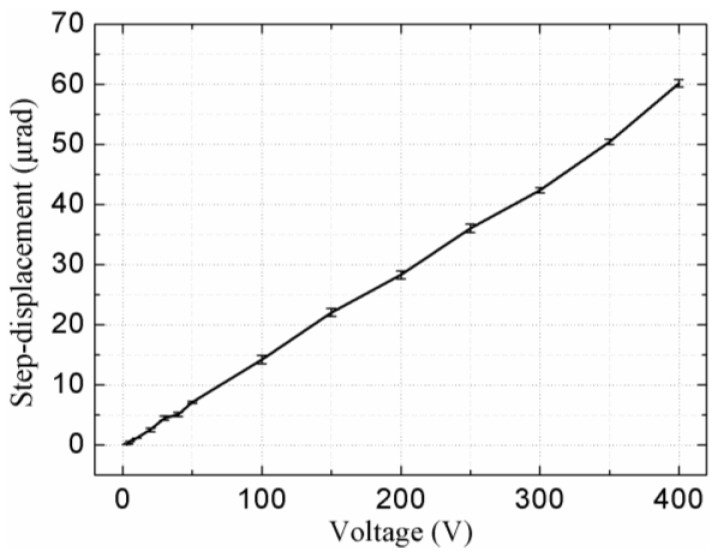
Plot of the step-displacement versus the voltage applied to the PZT-H of the vibrators.

**Figure 11:**
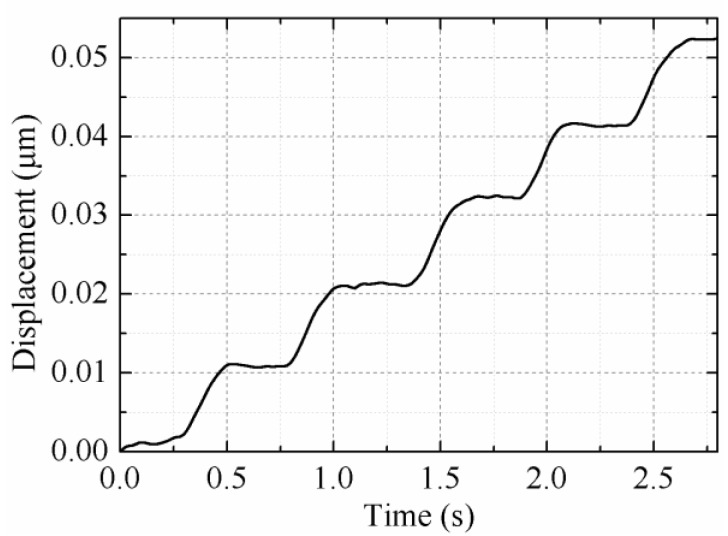
Plot of the displacement under voltage with step increment of 3 V_p-p_.

The step-displacement is shown in Figure 10, it was tested when the signals applied to PZT-V were fixed at 400 V_p-p_ and the signals applied to PZT-H were changed. The step distance is measured to be about 10.5 nm under voltage with step increment of 3 V_p-p_, as shown in Figure 11. The radius of the rotor is 110 mm; therefore, the minimum stable output displacement is calculated to be about 0.095 μrad, which is limited by the measurement noise, accuracy of the driving voltage, and the resolution of the capacitance displacement sensor. Theoretically speaking, the output displacement of the piezoelectric element is proportional to the applied voltage, thus, the piezoelectric actuator can achieve higher displacement resolution by using a power supply with higher output voltage resolution. A proper closed-loop control method can achieve higher positioning accuracy and the next step in this work is to implement the control-loop to enhance the system accuracy.

**Figure 12:**
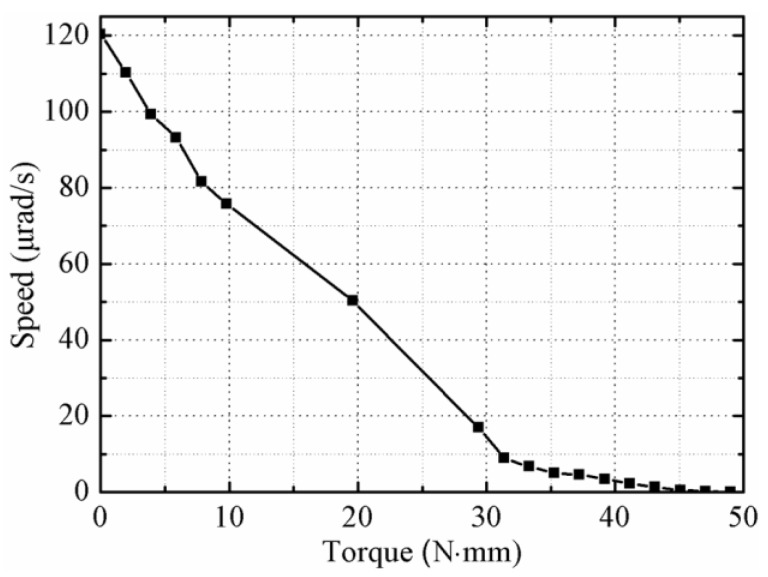
Plot of the speed versus the torque.

Finally, the mechanical property of the proposed actuator was tested under trapezoidal signals with a frequency of 1 Hz, an amplitude of 400 V_p-p_, and rise time of 5 ms, and the preload was still set as 12 N. The speed versus output torque curve is shown in Figure 12. It was found that the maximum no-load speed is 120.38 μrad/s and the maximum torque is 49 N·mm.

Table 4 shows the comparison between the proposed actuator and several existing rotary precision actuators of their presented excellent performances [18,19,20]. Although the velocity (steady state: no slip) and output torque that the proposed actuator can achieve are relatively lower, it exhibits several advantages as well. Firstly, the proposed actuator achieves the rotary driving with unlimited stroke by a new mechanism: four-feet walking. The structure of this actuator is relatively simple since it uses the bending vibrators, not PZT stacks. Furthermore, the rotary resolution of the proposed actuator is quite high, which means that it will be very valuable for a system with a high-precision requirement. In summary, the proposed actuator achieves simple structure, high resolution, and unlimited stroke. Additionally, the speed of the actuator can be varied by changing the driving voltage and working frequency.

## 5. Conclusions

A stepping piezoelectric actuator using a four-feet walking mechanism was proposed, designed, fabricated, and tested in this work for the aim of rotary driving with high resolution. The desired rotary stepping motion of the rotor was accomplished by the cooperation of four driving feet moved in rectangular trajectories, and two orthogonal bending motions were used to form the rectangular motion on each driving foot. The horizontal and vertical displacements of the driving foot were designed to be about 6.36 μm and 6.08 μm, respectively, by using a bolt-clamped vibrator with a total length of 80 mm. The transient response gained by the FEM stated that we could obtain different linear accelerations of the driving feet by using driving signals with different transitional times, and a rise time of 5 ms was determined to ensure that there were no slipping motions between the driving feet and the rotor. The experiments of the prototype proved the feasibility of the proposed mechanism: the rotor was moved step-by-step smoothly, a minimum resolution of 0.095 μrad and a maximum torque of 49 N·mm were achieved., The proposed actuator achieved rotary driving with unlimited stroke and high resolution by a new four-feet walking mechanism by comparing with the previous rotary piezoelectric actuators. Future work will focus on the development of a proper closed-loop control method.

## Figures and Tables

**Table 1:** The parameters of the PZT elements.

Parameters	Unit	Nomenclature	Value
Density	kg/m^3^	*ρ*	7600
Poisson’s ratio	×10^10^ N/m^2^	*σ^E^*	0.32
Elastic modulus		*c*_11_	14.3
		*c*_12_	7.85
		*c*_13_	7.85
		*c*_33_	11.5
		*c*_44_	2.6
		*c*_66_	2.45
Piezoelectric constants	C/m^2^	*e*_31_	−2.4
		*e*_33_	17.3
		*e*_15_	12.95
Relative dielectric constants		$\varepsilon_{11}^{S}/\varepsilon_{0}$	765
		$\varepsilon_{33}^{S}/\varepsilon_{0}$	640

**Table 2:** Maximum linear accelerations of driving foot under different signals.

Time Rise Time of Signal (ms)	a (m/s^2^)
0	1555
0.5	123.5
1	68.34
2	30.87
5	12.35

**Table 3:** The step-displacements of the four vibrators.

Vibrator No.	Step-Displacement (μm)
I	6.27
II	6.43
III	6.68
IV	6.55

**Table 4:** Comparisons of several rotary precision actuators.

Actuator	Mechanism	Structure	Resolution (μrad)	Stroke (μrad)
The proposed actuator	Walking	Clamped transducer	0.095	2π × 10^6^
The actuator by Clark et al. [18]	Direct driving	Flexure hinge	0.075	535.8
The actuator by Wang et al. [19]	Inertial driving	Flexure hinge	0.24	2π × 10^6^
The actuator by Li et al. [20]	Inertial driving	Flexure hinge	1.54	2π × 10^6^
